# A Comparative Analysis of the Properties of Coal Liquefaction Residues and Limestone Fine Aggregates

**DOI:** 10.3390/ma19101994

**Published:** 2026-05-12

**Authors:** Hao Wu, Zhe Wang, Pengfei Li, Mingliang Li, Jun Li, Shuangfeng Guo

**Affiliations:** 1 Research Institute of Highway Ministry of Transport, Beijing 100088, China; 2 Beijing Municipal Road & Bridge Building Material Group Co., Ltd., Beijing 100176, China; 3 Chongqing University Industrial Technology Research Institute, Chongqing 400030, China; 4 Beijing Xinqiao Technology Development Co., Ltd., Beijing 100088, China; lipengfei3060@163.com; 5 College of Transportation Engineering, Nanjing Tech University, Nanjing 210000, China

**Keywords:** direct coal liquefaction residue, indirect coal liquefaction residue, physical characteristics, chemical characteristics, fine aggregate

## Abstract

Coal liquefaction residues (CLRs), including both indirect (ICLR) and direct (DCLR) variants, represent industrial by-products whose conventional landfill disposal raises environmental concerns. This study comparatively analyzes ICLR and DCLR properties against limestone fine aggregates through physicochemical characterization. Results indicate that ICLR contains predominant SiO_2_ crystalline phases (50.05%) with trace Fe-Ti-Al-Mg oxides, demonstrating higher Vickers hardness (615 HV vs. 246 HV for limestone) and elastic modulus (98 GPa vs. 81 GPa for limestone), while its apparent relative density (2.612) closely matches that of limestone (2.783). Conversely, DCLR features abundant carbonaceous components (75.9% C) with olefinic/aromatic structures (asphaltene content 66.2%), exhibiting lower mechanical strength (Vickers hardness 21 HV) but enhanced asphalt affinity, as indicated by strong C=C (1591 cm^−1^) and aromatic C–H (744 cm^−1^) absorption peaks in FTIR. Both CLRs share comparable gradation curves and micromorphological characteristics with limestone aggregates, including uniform surface scaly textures. While pore-size distributions differ minimally between CLRs, both present finer porosity than limestone and show no leachate toxicity risks, confirming their viability as sustainable alternatives to asphalt fine aggregates.

## 1. Introduction

The energy landscape of China is notably characterized by its “abundance in coal and scarcity in oil and gas.” Coal maintains an unequivocal dominance in China’s primary energy supply, accounting for a substantial 70% share [1]. Despite the relentless enhancement of environmental standards and the burgeoning adoption of renewable energy sources, which have led to a modest decline in coal’s share of the energy mix, the reliance on coal remains deeply ingrained due to the nation’s energy resource endowment. Consequently, the predominance of coal in China’s energy production and consumption structure is not expected to undergo a radical transformation in the foreseeable future [2]. In pursuit of the “dual-carbon” target, the imperative for the clean and efficient utilization of coal has become more pressing than ever. Reflecting this urgency, China has prioritized the clean and efficient use of coal among the 100 key projects and initiatives outlined in its “14th Five-Year Plan” [3]. Coal liquefaction technology stands as a pivotal technical strategy for achieving this goal, facilitating the conversion of solid coal into liquid fuels, as well as chemical raw materials and products, through a series of chemical reactions. This advanced clean coal technology encompasses two distinct processes: direct and indirect liquefaction [4,5]. The direct liquefaction process involves the treatment of coal with hydrogen, a hydrogen-donating solvent, and a catalyst under extreme temperatures and pressures. This process, which includes thermal extraction, dissolution, and decomposition, transforms solid coal into liquid fuels and chemical raw materials. On the other hand, the indirect liquefaction process begins with coal gasification, followed by decarbonization and desulfurization, leading to the Fischer–Tropsch synthesis that produces a range of alkane and olefin compounds. Subsequent steps involve tail gas treatment and product refinement to yield high-quality oil and chemical products [6,7]. Nonetheless, as a by-product of these coal liquefaction processes, approximately 30% of the original coal material is converted into by-products—namely direct coal liquefaction residue (DCLR) and indirect coal liquefaction residue (ICLR) [8].

Economic growth has spurred an expansion in the scale of road construction and maintenance efforts in China. By the end of 2022, the nation’s operational road network had ballooned to an impressive 535 million kilometers, the largest in the world. Within this network, semi-rigid base asphalt pavements constitute over 90% of the road structures in China. However, the aging of asphalt leads to a progressive decline in road service performance, necessitating the construction and maintenance of nearly 400,000 km of roads annually. This demand consumes a staggering 50 billion tons of natural sand, gravel, and other materials [9]. In tandem with these developments, the Chinese government has been proactive in enacting policies aimed at fostering a diversified utilization of coal that mitigates environmental pollution. These efforts are designed to bolster the circular economy, enhance the efficient use of resources, and establish robust systems for recycling both resources and waste materials. The high-value conversion and utilization of DCLR and ICLR are particularly crucial for enhancing the efficiency and level of coal resource utilization and for the responsible management of these residues [10]. Yet, the current approaches to managing ICLR often involve simple piling or landfilling, which not only leads to environmental contamination but also squanders valuable land resources. DCLR, on the other hand, is typically subjected to combustion, coking, or cracking, methods that are not only limited in scope but also result in low utilization rates. The annual increase in DCLR and ICLR stands at 1 million tons and between 700 and 1000 million tons, respectively. Failure to address the proper treatment of such vast quantities of industrial solid waste could lead to escalating future risks [11].

To harness the full potential of coal liquefaction residues, researchers have been integrating them into road engineering applications. Studies have revealed that ICLR is characterized by a hard texture and a density comparable to that of natural sand and gravel, with the added benefit of being resistant to melting at high temperatures. Its primary constituents, including SiO_2_, CaO, Al_2_O_3_, and Fe_2_O_3_, suggest that ICLR could serve as either an aggregate or an admixture in road construction [12,13]. DCLR, on the other hand, comprises 20% to 30% heavy oil, 20% to 40% asphaltene, 15% to 30% pre-asphaltene, and approximately 45% tetrahydrofuran-insoluble substances. This composition endows DCLR with the versatility to function not only as a modifier for road petroleum asphalt but also as a fine aggregate in asphalt mixtures. Its inclusion can significantly prevent pavement deformation and enhance the longevity of asphalt pavements [14]. Given the nascent stage of research into the use of coal liquefaction residues as fine aggregates, there is a paucity of in-depth studies on this topic. This article aims to bridge that gap by delving into the physical and chemical properties of coal liquefaction residues. Through a comprehensive analysis, it will assess the viability of incorporating these residues as fine aggregates in asphalt mixtures, offering a potential solution for sustainable road construction and waste management.

## 2. Physical Properties

### 2.1. Basic Performance

(1) Particle Composition

**Figure 1:**
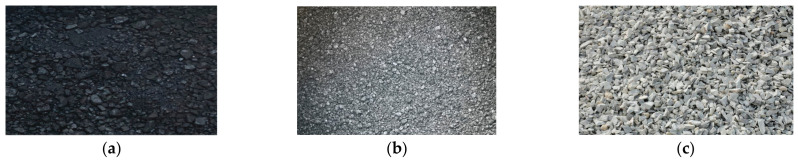
DCLR, ICLR and limestone. (**a**) DCLR, (**b**) ICLR, (**c**) limestone.

DCLR, a by-product derived from China Shenhua Coal-to-Oil Chemical Co., Ltd., Ordos, China, is presented in Figure 1a. This black solid particle remains stable at room temperature and exhibits a melting point near 190 °C, undergoing a phase transition when subjected to high temperatures.

ICLR, sourced from the Shanxi Changzhi Lu’an Group and depicted in Figure 1b, is a black-brown solid particle that retains its solid state at room temperature. Distinct from DCLR, ICLR does not melt under high temperatures and is composed of residual catalysts, alkane substances, and metal oxides.

The limestone used in this study is extracted from the Fangshan Quarry, Beijing, China, as shown in Figure 1c. This material is categorized into three distinct particle size ranges: 9.5~20 mm, 4.75~9.5 mm, and 0~4.75 mm. The fine mineral powder is obtained through the grinding process of limestone, ensuring a uniform and fine aggregate for use in various applications.

**Figure 2:**
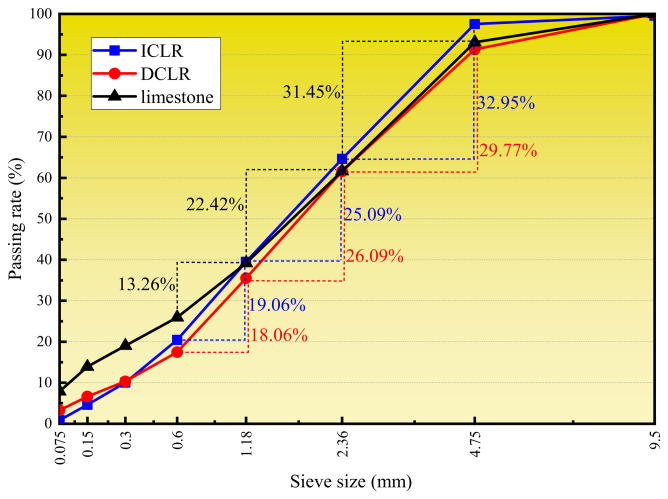
Screening curve of ICLR, DCLR and limestone fine aggregate [15].

The ICLR and DCLR were dedusted, broken and screened, and the particle size distribution was compared with that of limestone fine aggregate. The particle size distribution of the three materials is shown in Figure 2.

Figure 2 shows that the particle compositions of ICLR and DCLR closely resemble those of limestone fine aggregate. The particle size distributions for all three are 0.6~4.75 mm, accounting for 77.10%, 73.91%, and 67.13%, respectively. While the cumulative sieve residues across the four critical sieve sizes (0.075 mm, 1.18 mm, 2.36 mm, and 4.75 mm) are similar, there are variations in the particle proportions below 0.075 mm, at 0.9%, 3.3%, and 7.9%, respectively, due to the high mud content in limestone fine aggregate and dust in the fine aggregate. The 1.18 mm sieve proportions are 25.09%, 26.09%, and 22.42%, and the 2.36 mm sieve proportions are 32.95%, 29.77%, and 31.45%, with minimal differences among them. Above 4.75 mm, the proportions are 2.45%, 8.63%, and 6.93%, with DCLR being more similar to limestone fine aggregate and ICLR having the smallest proportion.

(2) Technical Indicators

The ICLR, DCLR and limestone fine aggregate were evaluated according to the technical standards of fine aggregate in “Technical Specifications for Construction of Highway Asphalt Pavements” (JTG F40—2004) [16]. All key performance indicators (density, sand equivalent, moisture content, etc.) are presented as averages based on the results of at least three parallel tests to ensure the reliability of the data. The results are shown in Table 1 [17].

(1) Table 1 shows that ICLR and limestone fine aggregate have similar apparent relative density and solidity. Both have low mud content, though limestone fine aggregate is slightly higher. ICLR has a higher sand equivalent, indicating better cleanliness, as limestone fine aggregate may contain impurities like soil and dust, while ICLR mainly contains unreacted catalysts with relatively simple impurities. ICLR is slightly less angular than limestone due to its smoother surface and lower inter-particle friction.
(2) DCLR exhibits an apparent relative density approximately half that of limestone fine aggregate, accompanied by slightly lower solidity. In terms of mud content and sand equivalent, however, the differences between the two materials are negligible. Notably, DCLR demonstrates higher angularity than limestone fine aggregate; this is primarily attributed to its low density, light particle mass, and poor fluidity. Regarding water absorption, limestone fine aggregate shows a value of 1.2%, compared to 1.5% for ICLR and 1.9% for DCLR. The elevated water absorption of DCLR is mainly due to its high organic content and porous structure. Consequently, in practical applications, this characteristic necessitates appropriate adjustments to the asphalt content or the implementation of pre-mixing treatments.
(3) Compared with ICLR and limestone fine aggregate, DCLR melts at a relatively high temperature. Therefore, according to the technical standards related to asphalt performance in “Test Methods for Asphalt and Asphalt Mixtures for Highway Engineering” (JTG E20—2011) [18], basic performance tests were carried out on DCLR, and the technical standards of 90# asphalt were selected for comparison, as shown in Table 2. All key performance indicators are presented as averages based on the results of at least three parallel tests to ensure the reliability of the data.

Table 2 shows that the penetration and ductility of DCLR are relatively small, less than one-tenth of the technical indicators of 90# asphalt, indicating that DCLR has high hardness, low ductility, and high brittleness. The softening point is nearly five times that of 90# asphalt technical standards, indicating that DCLR has strong heat resistance compared to ordinary asphalt.

### 2.2. Mechanical Properties

The MH-500 type micro-Vickers hardness tester and the Hysitron TI 950 type nanoindenter from the United States were used to test the Vickers hardness and elastic modulus of ICLR, DCLR and limestone fine aggregate, and the results are shown in Table 3. All key performance indicators are presented as averages based on the results of at least three parallel tests to ensure the reliability of the data.

(1) Table 3 illustrates that the Vickers hardness of ICLR is 2.5 times greater than that of limestone fine aggregate, and its modulus of elasticity is 1.2 times greater. Despite having a slightly lower density compared to limestone fine aggregate, ICLR emerges as a lightweight, high-strength material. It exhibits significantly higher hardness and modulus, as well as superior compressive and destructive resistance compared to limestone fine aggregate.
(2) DCLR exhibits Vickers hardness and elastic modulus values that are merely one-tenth those of limestone fine aggregate, indicating its intrinsically inferior hardness and deformation resistance. This marked discrepancy stems from the disordered arrangement and weak cohesion among DCLR’s organic components. In contrast, limestone fine aggregate possesses an ordered, tightly bonded molecular structure, typically characterized by strong metallic or ionic bonds that underpin its superior hardness and deformation resistance.

### 2.3. Microscopic Morphology

**Figure 3:**
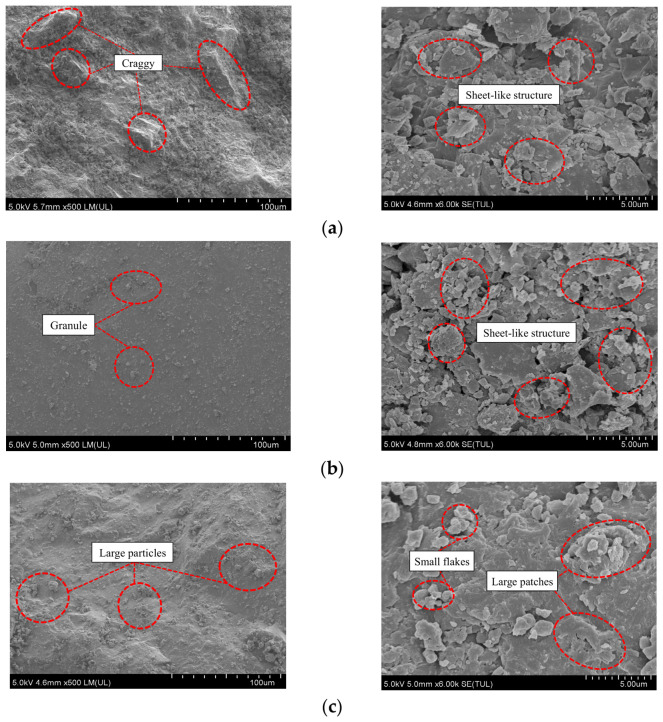
Microscopic morphology of ICLR, DCLR and limestone fine aggregates [19]. (**a**) Limestone fine aggregate, (**b**) ICLR, (**c**) DCLR.

The microscopic morphology of ICLR, DCLR, and limestone fine aggregate was examined using a German SU8020 scanning electron microscope at magnifications of 500× and 6000×, as shown in Figure 3 [19].

(1) Under 500× magnification (Figure 3), limestone fine aggregate shows pronounced angularity, unevenness, and a distinct pore structure with continuous, uniform peaks and valleys. ICLR and DCLR particles mainly adhere to irregular fine particle clusters of relatively uniform shape and size. ICLR is flatter than DCLR, with less surface undulation and a knitted flocculent structure; much of its surface consists of amorphous residual carbon, with numerous amorphous spherical glass phases clustered on it. In contrast, DCLR has a corrugated shape with consistent corrugation direction, densely packed granular material, and a denser melt from particle clusters than ICLR. Most small DCLR particles form plaque clusters with inconspicuous surface pores and contain uniformly attached residual carbon.
(2) At 6000× magnification, limestone fine aggregate, ICLR, and DCLR all exhibit similar continuous laminar scale-like structures. Limestone and ICLR show greater similarity in densification, forming a uniform, highly dense, cross-layered patchy lamellar structure, with higher fragmentation and arrangement density than DCLR. In contrast, DCLR possesses a denser surface than limestone but features a relatively sparse scale-like structure dominated by small lamellae that differ markedly from the larger lamellae and are unevenly distributed across them. Overall, the micro-morphologies of ICLR and DCLR are not significantly different from those of limestone; all exhibit a degree of surface roughness, which enhances asphalt adsorption and contributes to higher strength. ICLR and DCLR thus share certain micromorphological similarities with limestone.

### 2.4. Pore Size Distribution

**Figure 4:**
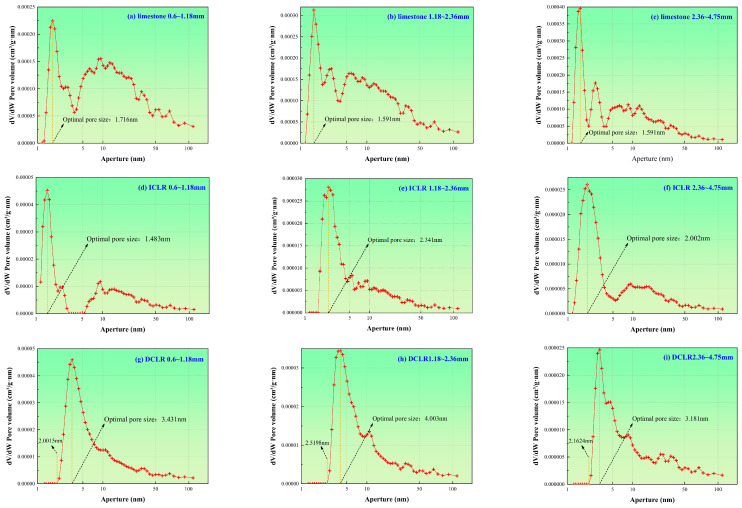
Pore size distribution [19]. (**a**) limestone 0.6–1.18 mm, (**b**) limestone 1.18–2.36 mm, (**c**) limestone 2.36–4.75 mm, (**d**) ICLR 0.6–1.18 mm, (**e**) ICLR 1.18–2.36 mm, (**f**) ICLR 2.36–4.75 mm, (**g**) DCLR 0.6–1.18 mm, (**h**) DCLR 1.18–2.36 mm, (**i**) DCLR 2.36–4.75 mm.

The pore size distribution of ICLR, DCLR, and limestone fine aggregate in three particle size fractions (0.6–1.18 mm, 1.18–2.36 mm, and 2.36–4.75 mm) was determined using a Japanese BELSORP-max fully automatic specific surface and pore analyzer, as illustrated in Figure 4 [19].

(1) According to Figure 4, the most probable pore diameter of ICLR is larger than that of limestone fine aggregate. Across the three particle sizes, both materials exhibit similar pore size distributions, primarily consisting of mesopores in the 2–50 nm range, along with minor amounts of micropores (<2 nm) and macropores (>50 nm). In contrast, DCLR shows a larger most probable pore diameter than both limestone fine aggregate and ICLR, indicating a more developed pore structure in the larger size range.
(2) Across three ICLR particle sizes, micropore volume and number vary little, with maximum pore volume concentrated between 0.000025 and 0.000045 cm^3^/g·nm. Mesopore and macropore volumes for the 1.18–2.36 mm and 2.36–4.75 mm sizes are greater than for the 0.6–1.18 mm sizes, and generally increase with particle size, implying greater asphalt accommodation. In contrast, limestone fine aggregate has a micropore void volume of 0.002–0.004 cm^3^/g·nm; its meso- and macropore structures for 0.6–1.18 mm and 1.18–2.36 mm are similar and higher than for 2.36–4.95 mm. Overall, limestone’s total pore volume exceeds that of ICLR.
(3) The pore diameter distributions for the three particle sizes of DCLR are all above 2 nm, indicating the absence of micropore structures and the presence of only mesopores and macropores. The maximum pore volume of DCLR’s pore diameter distribution is similar to that of ICLR, concentrated between 0.000025 and 0.000045 cm^3^/g·nm. For mesopores of 2 to 50 nm and macropores larger than 50 nm, the pore diameter distributions and corresponding void volumes of the 1.18 to 2.36 mm and 2.36 to 4.75 mm particle sizes of DCLR are nearly identical, suggesting minimal differences in the pore diameter distribution across the three particle sizes. Comparatively, the overall pore volume of limestone fine aggregate remains larger than that of DCLR from a quantitative standpoint.

## 3. Chemical Characteristics

### 3.1. Elemental Composition

The elemental composition of ICLR, DCLR, and limestone fine aggregate was determined using a German Elementar Vario EL organic elemental analyzer in conjunction with an inductively coupled plasma (ICP) spectrometer from Germany. The results are summarized in Table 4 [19]. To guarantee data reliability, all reported values represent the average of at least three parallel measurements.

(1) As shown in Table 4, limestone fine aggregate consists of four main elements. Carbon (C), oxygen (O), and calcium (Ca) are the dominant components, together making up more than 98% of the total composition, while silicon (Si) is present only in a trace amount.
(2) ICLR mainly consists of O (49.43%), Si (14.84%), C (11.44%), and S (7.63%), along with smaller proportions of Al (6.11%) and Ca (5.17%). Other elements detected include non-metals H and N, as well as metals Fe, Ti, and Mg. Relative to limestone fine aggregate, ICLR exhibits a much richer variety of both non-metal and metal elements, indicating good potential for use as an aggregate material.
(3) The elemental composition of DCLR is dominated by carbon, hydrogen, nitrogen, and sulfur, with carbon and hydrogen occurring in relatively high proportions. The elevated C/H atomic ratio points to an abundance of aromatic rings in its molecular structure. Within DCLR, polar heteroatom-containing groups (S, N, and O) are embedded in condensed aromatic matrices through non-covalent interactions such as hydrogen bonding, π-H bonding, and π-π stacking [20]. Carbon atoms are present mainly as benzene rings, methyl groups, and cyclic methylene units; oxygen occurs chiefly as carbonyl and ester functionalities, while nitrogen is largely incorporated in pyrrole rings [21].

### 3.2. Molecular Weight Distribution

**Figure 5:**
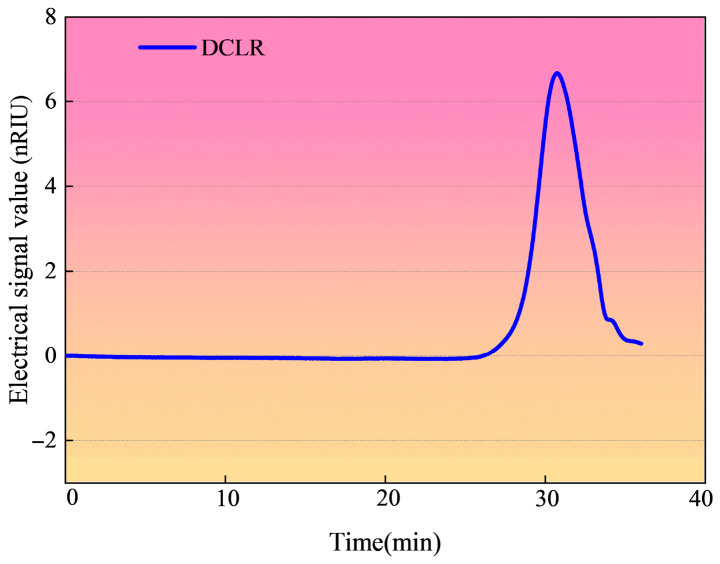
Molecular weight distribution of DCLR.

Unlike ICLR and limestone fine aggregate, DCLR is soluble in organic solvents such as tetrahydrofuran (THF). The molecular weight of DCLR can be ascertained only if it is part of the THF phase system, the aqueous phase system, or the dimethylformamide (DMF) phase system, that is, when the material is solubilized in the mobile phase. Consequently, the molecular weight distribution of DCLR, as depicted in Figure 5, can be analyzed within these phase systems.

Figure 5 plots the weight-average molecular weight of DCLR as 497, with a polydispersity index of 1.46 and an average molecular formula of C25H31O0.2N0.26. These characteristics suggest that DCLR is a small-molecule substance [22]. DCLR is predominantly composed of non-polar organic matter, specifically condensed polycyclic aromatic hydrocarbons containing 4 to 7 rings, with the condensed aromatic rings of these large molecules constituting the primary structural units. The pyridine-soluble fraction of DCLR contains 4 to 5 aromatic ring structures, and these structural units are adorned with methyl, hydroxyl, and cycloalkane groups [23]. During the heating process, the viscosity of DCLR decreases rapidly, without any viscosity peak, indicating its behavior as a non-Newtonian pseudoplastic fluid. At high temperatures, DCLR’s flow behavior approaches that of a Newtonian fluid.

### 3.3. Chemical Composition

An X-ray fluorescence spectrometer (XRF) of German S2 PUMA was used to test the oxide content of ICLR and limestone fine aggregate, and DCLR was analyzed for its composition in accordance with the four-component test method for asphalt and the chemical extraction method, and the results are shown in Table 5, Table 6 [15] and Table 7 [15]. All key performance indicators are presented as averages based on the results of at least three parallel tests to ensure the reliability of the data.

(1) Table 5, Table 6 and Table 7 indicate that the primary constituents of ICLR are SiO_2_, Al_2_O_3_, and CaO, with significant amounts of Fe_2_O_3_, TiO_2_, and MgO also present. In comparison to limestone fine aggregate, ICLR has a higher SiO_2_ content, whereas limestone fine aggregate contains only 2.4% SiO_2_. Chemically, SiO_2_, being an atomic crystal with covalent bonds, requires significantly more energy to break its bonds compared to the ionic bonds in CaCO_3_, an ionic crystal. Consequently, SiO_2_ is harder than CaCO_3_, implying that ICLR is harder than limestone fine aggregate.
(2) DCLR can be fractionated into four components through sequential extraction with distinct solvents (n-hexane, toluene, and tetrahydrofuran): heavy oil, asphaltene, pre-asphaltene, and tetrahydrofuran-insoluble substances. The mass fractions are as follows: heavy oil (20–30%), primarily naphthalene derivatives with alkyl substitutions; asphaltene (20–40%), mainly condensed aromatic hydrocarbons with six-membered rings; pre-asphaltene (15–30%), composed of multiple condensed aromatic hydrocarbons linked by bridge bonds and hydrogenated aromatic hydrocarbons; and THFIS, primarily unreacted coal, quartz, calcium sulfate, pyrite, and other minerals, with a mass fraction of about 45%. According to the classification of asphalt into four components, DCLR can also be categorized into saturates, aromatics, asphaltenes, and resins. The low content of saturates and aromatics contributes to DCLR’s high viscosity, while the low resin content results in poor adhesion and ductility in macroscopic performance. The high asphaltene content leads to a high softening point and increased viscosity, endowing DCLR with hard, brittle, and viscous characteristics.
(3) The primary component of DCLR is organic matter, predominantly asphaltenes at 66.20%, followed by resins at 13.91%, with trace amounts of saturates and aromatics. Additionally, DCLR contains about 15% ash, which is inorganic matter. This indicates that DCLR is composed of approximately 85% organic matter and 15% inorganic matter. Although DCLR is not primarily composed of oxides like ICLR or limestone fine aggregate, the organic compounds it contains provide a favorable material basis for enhancing the adhesion between asphalt and aggregates, increasing the bonding strength of asphalt binders, improving the elasticity, and reducing the cracking of the mixture.

### 3.4. Crystal Phase

**Figure 6:**
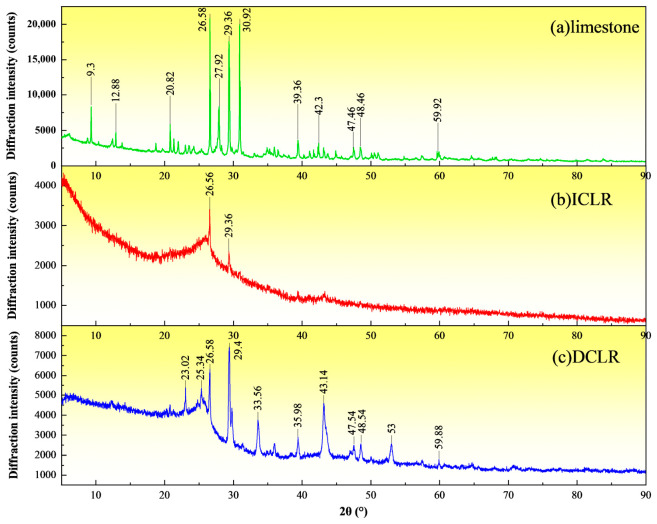
XRD spectrum of ICLR, DCLR and limestone fine aggregate. (**a**) limestone, (**b**) ICLR, (**c**) DCLR.

X-ray diffraction (XRD) tests were carried out on three materials, ICLR, DCLR and limestone fine aggregate, using a Rigaku SmartLab SE instrument from Japan, under the following conditions: scanning angle of 5–90°, scanning speed of 2°/min, step size of 0.02°, ray source Cu K-Alpha1, wavelength of 1.540598 Å, voltage of 40 KV, tube current of 40 mA. The results are shown in Figure 6.

(1) Figure 6 reveals that, in accordance with the Powder Diffraction File (PDF) standards, the limestone fine aggregate contains various crystalline phases. Specifically, a diffraction peak at 9.3° suggests the presence of trace laumontite crystals (Ca[AlSi_2_O_6_]_2_·4H_2_O). Peaks at 26.58° and 27.92° are attributed to quartz (SiO_2_) and sphene (CaTi[SiO_4_]O) crystals, respectively, while the peak at 29.32° is indicative of calcite (CaCO_3_). The predominant phase in the limestone fine aggregate is calcite (CaCO_3_), with quartz (SiO_2_) as a secondary phase. Elements such as magnesium (Mg), aluminum (Al), and iron (Fe) are typically found in the form of oxides within the calcium carbonate matrix. Traces of anorthite (CaSiO_3_) and wollastonite (CaSiO_3_) are also detected [24].
(2) The X-ray diffraction (XRD) pattern of ICLR exhibits a broad, upward-protruding peak between 20° and 30°, colloquially referred to as a “steamed bun peak,” which is indicative of non-crystalline compound aggregation. Specific peaks are identified at 26.56° for quartz (SiO_2_) and at 29.36° for calcite. ICLR is characterized by a scarcity of distinct diffraction peaks, with the predominant phase being an amorphous glassy matrix, predominantly aluminosilicate, accompanied by minor crystalline phases such as quartz (SiO_2_), mullite (3Al_2_O_3_·2SiO_2_), calcite, and hematite (Fe_2_O_3_). The amorphous components include glass, slag wax, and amorphous carbon [25]. The original crystalline structure of inorganic minerals is altered to a glassy state following intense high-temperature oxidation, resulting in small and flat crystal peaks in the XRD pattern [26]. Quartz, due to its high melting point, retains some crystalline structure in parts that have not melted. ICLR thus primarily presents an irregular glassy structure, composed predominantly of amorphous materials with a minor presence of crystalline substances.
(3) In the small-angle region (2θ ≤ 13°), the diffraction peaks of DCLR exhibit a monotonically increasing trend. As documented in references [27,28], the peaks within the range of 2θ ≤ 35° are predominantly a result of the superposition of two distinct microcrystalline diffraction peaks: the 002 peak and the γ peak. The 002 peak, which occurs between 20° and 35°, arises from carbon microcrystals formed by the stacking of aromatic layers, also referred to as aromatic carbon microcrystals. This peak reflects the parallel and azimuthal alignment of aromatic carbon layers in three-dimensional space; a taller and narrower peak suggests a more ordered arrangement of these layers. Conversely, a broader 002 peak is indicative of the intercalation of small-sized microcrystals between the aromatic layers. The γ peak, observed at angles less than or equal to 20° (2θ ≤ 20°), is attributed to aliphatic hydrocarbon side chains, diverse functional groups, and carbon microcrystals associated with the aliphatic hydrocarbons that are linked to the aromatic layer structure, collectively termed aliphatic carbon microcrystals.
(4) The XRD peak intensities for limestone fine aggregate range from 5000 to 22,500, for ICLR from 2500 to 3500, and for DCLR from 2000 to 7000. Relative to DCLR and ICLR, the limestone fine aggregate exhibits higher peak intensities and sharper peak shapes, signifying superior phase crystallinity and greater crystal content. DCLR demonstrates peak sizes and sharpness that exceed those of ICLR, suggesting that DCLR possesses higher crystallinity and a greater quantity of crystalline material compared to ICLR.
(5) Collectively, the concentrations of Ca, Mg, and Fe oxides in ICLR and DCLR are lower than those found in limestone fine aggregate, resulting in a generally lower alkalinity for these aggregates when compared to limestone fine aggregate. Nonetheless, ICLR contains minor quantities of Ca, Mg, and Fe oxides and exhibits a higher alkalinity than DCLR. Consequently, considering the aspects of mineral crystal phases and material properties, ICLR is deemed more appropriate as a fine aggregate material.

### 3.5. Functional Groups

**Figure 7:**
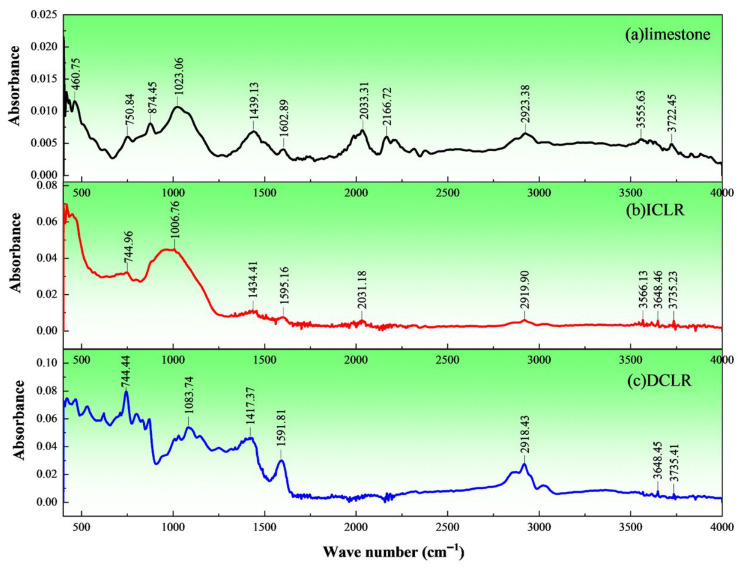
Fourier transform infrared spectrometer of ICLR, DCLR and limestone fine aggregate. (**a**) limestone, (**b**) ICLR, (**c**) DCLR.

Fourier transform infrared (FTIR) spectroscopy was carried out on the three materials of ICLR, DCLR and limestone fine aggregate by using the American Thermo Scientific Nicolet iS20 instrument with the following conditions: scanning range of 350~7800 cm^−1^, moving mirror rate of 0.47 cm/s, 32 scans, 4 cm^−1^ resolution, 50,000:1 signal-to-noise ratio. The results are shown in Figure 7.

(1) Figure 7 shows three main fingerprint-region peaks (400–1300 cm^−1^) for limestone fine aggregate: a minor one-substituted benzene ring peak at ~750.84 cm^−1^, a moderate para-disubstituted benzene ring peak at ~874.45 cm^−1^, and an intense carbonyl double peak at 1023.06 cm^−1^. In the functional group region (1300–4000 cm^−1^), 3–5 peaks of similar intensity appear: at 1439.13 cm^−1^ (-CH_3_), 2166.72 cm^−1^ (unsaturated R_2_C=CHR), and a broader, weaker peak at 2923.38 cm^−1^ (-CH_2_-). This indicates that the aggregate contains significant alkane substances.
(2) In the fingerprint region, ICLR shows two main peaks: a one-substituted benzene peak at 744.96 cm^−1^ and an intense, broad C-OH hydroxyl peak at 1006.76 cm^−1^. In the functional group region, 3–5 peaks of comparably lower intensity appear: -CH_3_ bending at 1434.41 cm^−1^, C=C stretching near 1595.16 cm^−1^, a minor -CH_2_- peak near 2919.90 cm^−1^, and a medium free O-H hydroxyl stretch at 3648.46 cm^−1^. These features indicate ICLR contains alkanes, free secondary amines (amine compounds), alcohols and phenolics.
(3) In the functional group region (1300–4000 cm^−1^), DCLR shows three main peaks: a medium C=C absorption near 1591.81 cm^−1^, stretching vibrations for -CH_3_ and -CH_2_- near 2918.43 and 2850 cm^−1^, and an O-H stretch between 3600 and 3700 cm^−1^. In the fingerprint region (400–1300 cm^−1^), DCLR has more characteristic peaks, which are broader and more dispersed, mainly between 1000 and 1300 cm^−1^ and 500–800 cm^−1^. A sharp, narrow peak at 744.44 cm^−1^ indicates a one-substituted benzene structure, and a medium broad peak at 1083.74 cm^−1^ indicates C-OH groups. Collectively, these features suggest DCLR is an unsaturated hydrocarbon compound, specifically an alkane-substituted benzene isomer. In the FTIR spectrum of DCLR, the absorption peak at 1591 cm^−1^ corresponds to the C=C stretching vibration of the aromatic ring; the sharp peak at 744 cm^−1^ corresponds to the monosubstituted benzene ring structure; and the broad peak at 1083 cm^−1^ corresponds to the C–OH hydroxyl stretching vibration. Among these functional groups, the aromatic ring structure forms π–π conjugation with the aromatic components in asphalt, serving as the primary chemical mechanism for enhancing asphalt–aggregate adhesion; meanwhile, polar groups such as the C–OH hydroxyl group can form hydrogen bonds or acid–base interactions with polar sites on the aggregate surface (e.g., Ca^2+^, Si–OH), further improving interfacial bonding.
(4) Compared to ICLR and DCLR, limestone fine aggregate has more continuous and uniform characteristic peaks in both position and intensity. ICLR and DCLR show greater peak discontinuities, unevenness, and more pronounced intensity differences. Stronger, broader peaks are mainly in the fingerprint region, while weaker, narrower ones are in the functional group region. Absorbance values: limestone 0.005–0.01; ICLR strong peaks 0.03–0.05 (others ~0.005); DCLR strong peaks 0.03–0.08 (weak peaks 0.01–0.02). This indicates DCLR has the most diverse and concentrated functional groups, while ICLR and limestone show less significant overall differences.

### 3.6. Thermal Stability

**Figure 8:**
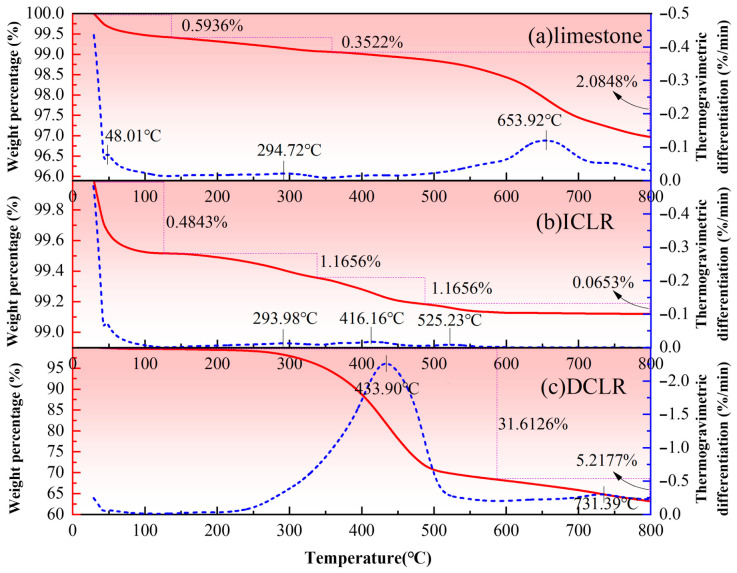
Thermogravimetric analysis of ICLR, DCLR and limestone fine aggregate. (**a**) limestone, (**b**) ICLR, (**c**) DCLR.

Thermogravimetric analysis of the three materials, ICLR, DCLR and limestone fine aggregate, was carried out using a German STA 449 F5 thermo-synchronous analyzer and the results are shown in Figure 8.

(1) It was found in Figure 8 that the weight of the three materials under study decreases with rising temperature. The weight loss of limestone fine aggregate occurs in three distinct stages: 0~131 °C, 131~353 °C, and 353~800 °C. For ICLR, the weight loss is distributed across four stages: 0~132 °C, 132~338 °C, 33~480 °C, and 480~800 °C. DCLR exhibits weight loss in two stages: 0~588 °C and 588~800 °C. The weight percentage is calculated as the ratio of the material’s weight at any given temperature to its initial weight. It is observed that from the initial temperature to 800 °C, ICLR experiences the least weight loss among the three materials, followed by limestone fine aggregate, and then DCLR. Consequently, the thermal stability ranking is ICLR (with the highest stability), limestone fine aggregate (in the middle), and DCLR (with the lowest). This is attributed to the fact that ICLR is primarily composed of complex oxides with a stable crystal phase and excellent high-temperature resistance. The main component of limestone fine aggregate is CaCO_3_, which maintains good thermal stability at lower temperatures but decomposes into CaO and CO_2_ at higher temperatures, resulting in weight loss. The significant weight loss of DCLR is primarily due to the volatilization of components such as asphaltenes and resins at elevated temperatures.
(2) Thermogravimetric differentiation, which involves taking the first derivative of the weight percentage curve with respect to time, characterizes the rate of weight change, with peak points indicating the temperatures at which the rate of weight change is most rapid during each stage. Analysis reveals that the limestone fine aggregate experiences its most rapid weight loss near 48 °C, 295 °C, and 654 °C. Prior to 500 °C, the limestone fine aggregate consistently exhibits a lower and more stable rate of weight loss, with an acceleration in mass loss beyond 500 °C. For ICLR, the most rapid weight loss occurs near 294 °C, 416 °C, and 525 °C, maintaining a lower and more stable rate of loss across the entire temperature range. DCLR’s most rapid weight loss is observed near 434 °C and 731 °C. Below 250 °C, DCLR shows a minimal weight loss rate, which begins to increase thereafter. The temperature range from 250 °C to 500 °C marks the interval of most rapid weight loss for DCLR, with the rate fluctuating little after 500 °C. However, compared to the initial range of 0–250 °C, DCLR still exhibits a higher rate of mass loss.
(3) DCLR is prone to melting and decomposition at elevated temperatures. Thermogravimetric analysis indicates that DCLR maintains relatively good thermal stability up to 250 °C. In road engineering, the preparation temperature for asphalt mixtures typically ranges from 160 °C to 180 °C and seldom exceeds 200 °C; even at higher discharge temperatures, it is around 240 °C. To investigate whether DCLR undergoes melting and decomposition within the mixer during asphalt mixture preparation, and consequently, whether there is a change in the proportion of its constituent components, we selected a test temperature range of 160 °C to 240 °C. The specific procedure involved setting four temperatures: 170 °C, 190 °C, 210 °C, and 230 °C. DCLR was placed in a muffle furnace and heated for 2 h at each of these temperatures. Subsequently, samples were taken for an asphalt four-component test to assess the changes in the four-component content of DCLR at these temperatures. The findings are presented in Table 8.

(1) Table 8 indicates that as the temperature is elevated from 170 °C to 230 °C, the loss on ignition of DCLR increases from 0.26% to 4.83%. This suggests that the degree of component loss in DCLR escalates with increasing temperature. The colloidal instability coefficient (I_c_) serves as a metric for assessing the type of asphalt colloidal structure and determining if the four components are within an optimal range. A higher I_c_ value signifies a less stable colloidal structure type. The data in the table reveal that with the temperature increase, the I_c_ of DCLR progressively diminishes, suggesting that the colloidal structure of DCLR becomes more stable. In other words, higher temperatures favor the attainment of a more stable colloidal structure for DCLR.
(2) With the increase in temperature, there is a gradual increment in the relative content of saturates, aromatics, and resins, while the relative content of asphaltenes decreases. This suggests that the reduction in asphaltenes is more pronounced than that of saturates, aromatics, and resins as the temperature rises. Specifically, within the temperature range of 170 °C to 230 °C, the loss of asphaltenes is 2.8%, whereas the losses for saturates, aromatics, and resins are 0.17%, 0.44%, and 1.16%, respectively. These findings imply that the incorporation of DCLR as a fine aggregate in mixture preparation may facilitate the penetration of its organic components into the asphalt, potentially inducing an asphalt modification effect.

### 3.7. Environmental Characteristics

To further verify the environmental safety of ICLR and DCLR as fine aggregates in road engineering, this study conducted leaching toxicity tests on two types of coal liquefaction residues and limestone fine aggregates in accordance with the “Solid Waste—Leaching Toxicity—Leaching Methods—Horizontal Shaking Method” (HJ 557-2010) [30]. Furthermore, in accordance with the “Criteria for the Identification of Hazardous Wastes—Identification of Leaching Toxicity” (GB 5085.3-2007) [31], quantitative analyses were performed on characteristic pollutants such as heavy metals and polycyclic aromatic hydrocarbons (PAHs) in the leachate. The test results are shown in Table 9.

The test results indicate that, with regard to heavy metal parameters, the concentrations of elements such as copper, lead, zinc, arsenic, mercury, cadmium, chromium, and nickel in the ICLR and DCLR leachates did not exceed the leaching toxicity identification limits specified in GB 5085.3-2007 [31]. Some heavy metal elements (such as lead, cadmium, and mercury) were not detected or were present only in trace amounts. Regarding organic pollutants, 16 typical polycyclic aromatic hydrocarbons (PAHs), including naphthalene, benzo[a]anthracene, benzo[a]pyrene, and indeno [3,4-c]pyrene, were not detected. This indicates that the organic components in both types of residues exhibit high structural stability under ambient temperature leaching conditions and are not easily transferred into the aqueous phase.

It is worth noting that although DCLR contains a high proportion of organic matter (approximately 85%), its polycyclic aromatic hydrocarbons primarily exist as fused aromatic ring structures. These compounds have high molecular weights and low polarity, resulting in extremely poor solubility in the aqueous phase. Additionally, the leaching tests used deionized water as the extraction medium, with a pH close to neutral, which further limited the dissolution of organic components. ICLR, on the other hand, consists primarily of inorganic oxides (such as SiO_2_ and Al_2_O_3_). Its glassy structure exhibits excellent chemical inertness at room temperature, with heavy metal elements stably encapsulated within the crystal lattice or glass phase, making them difficult to leach out.

Based on the comprehensive test results, no toxic or hazardous substances exceeding regulatory limits were detected in the leachate from ICLR and DCLR under simulated natural rainfall or surface water contact conditions (room temperature, neutral pH, solid-to-liquid ratio of 1:10). Compared to traditional disposal methods such as landfilling or open-air stockpiling, using these materials as fine aggregates in asphalt mixtures achieves both physical containment and asphalt encapsulation of solid waste, further reducing the risk of environmental exposure. Therefore, from the perspective of leaching toxicity, neither ICLR nor DCLR exhibits hazardous waste characteristics. When used as road construction materials, they pose no risk of contaminating the surrounding soil or groundwater environment and demonstrate good environmental safety.

## 4. Comparison of Physical and Chemical Properties

The results of the comparison of physicochemical properties of ICLR, DCLR and limestone fine aggregate are shown in Table 10.

Table 10 reveals that ICLR and DCLR share similar particle compositions, microscopic morphologies, and pore size distributions with limestone fine aggregate, and neither exhibits leaching toxicity. ICLR’s elemental composition and constituent components are akin to those of limestone fine aggregate, with a notable presence of SiO_2_ crystal structures, resulting in increased modulus and hardness. These characteristics suggest that ICLR is a viable candidate for use as a fine aggregate. DCLR, on the other hand, contains aromatic and polycyclic aromatic structures that readily interact with asphalt, enhancing the bonding strength of the asphalt binder. Given that DCLR’s physical properties closely resemble those of limestone fine aggregate, it too possesses the potential to be utilized as a fine aggregate to a certain extent.

## 5. Discussion

### 5.1. Comparison with Existing Literature

The SiO_2_ content of ICLR measured in this study was 50.05%, and its Vickers hardness (615 HV) was significantly higher than that of limestone (246 HV), consistent with the conclusions of Li et al. (2026) [32] regarding coal liquefaction residue as a high-strength aggregate.

The bituminous content of the DCLR in this study was 66.2%. The analysis and description of the C=C and aromatic ring characteristic peaks in the FTIR spectrum are consistent with the analysis of the aromatic structure of DCLR by Liu et al. (2025) [33]. Furthermore, DCLR exhibited a 2.8% loss of bituminous content at 170–230 °C, indicating that partial migration can occur at asphalt mixture mixing temperatures; this finding provides new evidence supporting the use of DCLR as an “active filler.”

In this study, neither heavy metals nor PAHs in the leachates of ICLR and DCLR exceeded regulatory limits, consistent with the evaluation results of Gao et al. (2024) [34] regarding the environmental safety of coal liquefaction residues, further validating their feasibility as green building materials.

### 5.2. Implications for Asphalt Pavement Applications

The hardness (615 HV) and elastic modulus (98 GPa) of ICLR are both superior to those of limestone, and its bulk density (2.612) is comparable, enabling it to enhance the compressive and shear resistance of asphalt mixtures without significantly increasing the pavement’s self-weight. Its high sand equivalent (93%) and low clay content (0.1%) also help ensure adhesion between the asphalt and aggregates [35].

Although DCLR has weaker mechanical properties (hardness of 21 HV), its rich aromatic structure and bituminous content allow it to partially soften during mixing and penetrate into the asphalt binder, creating an “in situ modification” effect. Given its high water absorption rate (1.9%), it is recommended to appropriately increase the asphalt content or adopt a pre-mixing process in practical applications.

ICLR and DCLR are highly similar to limestone in terms of gradation and microstructure; both can synergistically replace natural fine aggregates, achieving 100% substitution of solid waste [33].

Given that the Vickers hardness and elastic modulus of DCLR are significantly lower than those of limestone fine aggregate, it is not suitable for use as a primary load-bearing aggregate to serve as a structural framework. In practical applications, DCLR should be positioned as a functional filler or composite modifier, with its primary functions including forming a chemically compatible interface with asphalt through its high aromatic content to enhance asphalt-aggregate adhesion; allowing the lightweight components (saturated and aromatic fractions) in DCLR to partially migrate into the asphalt, creating an “in situ modification” effect that improves the flexibility and low-temperature crack resistance of the asphalt binder; and compensating for its relatively low mechanical properties by blending it with ICLR or limestone fine aggregate, forming a composite fine aggregate system that combines both rigidity and flexibility. Test results indicate that when the DCLR replacement rate is kept below 30%, both the dynamic stability and low-temperature flexural strain of the asphalt mixture meet code requirements.

## 6. Conclusions

(1) This study provides the first systematic comparative analysis of the physical and chemical properties of indirect coal liquefaction residue (ICLR) and direct coal liquefaction residue (DCLR) against limestone fine aggregate. It reveals that both ICLR and DCLR exhibit gradation compositions and microscopic morphological characteristics (uniformly distributed flaky surface structures) highly similar to those of limestone fine aggregate, with only minor differences in pore size distribution. These findings establish a fundamental material basis for substituting natural fine aggregates with coal liquefaction residues in asphalt mixtures.
(2) A key scientific contribution is the demonstration that ICLR, primarily composed of SiO_2_ crystalline phases and trace metal oxides (Fe, Ti, Al, Mg), possesses significantly higher Vickers hardness (615 HV) and elastic modulus (98 GPa) than limestone, along with superior thermal stability. Its technical specifications (apparent relative density, sand equivalent, and angularity) are comparable to those of limestone, making ICLR a high-strength, lightweight alternative that can enhance the compressive and shear resistance of asphalt pavements without increasing structural weight.
(3) For DCLR, the study reveals a unique “active filler” mechanism: rich in polycyclic aromatic hydrocarbons and asphaltenes (66.2%), DCLR exhibits lower mechanical strength but, within the typical asphalt mixing temperature range (170–230 °C), loses 2.8% of its asphaltene content along with partial saturates, aromatics, and resins. This controlled migration of organic components into the asphalt binder creates an in situ modification effect, improving asphalt–aggregate adhesion and mixture flexibility—a novel functionality not offered by conventional limestone fine aggregate.
(4) From an engineering and environmental perspective, both ICLR and DCLR pose no leaching toxicity risks, with heavy metals and polycyclic aromatic hydrocarbons remaining below regulatory limits. Their use as fine aggregates in pavement construction thus enables 100% substitution of natural aggregates, mitigates the environmental burden of landfill disposal, and supports circular economy goals in road infrastructure. These findings provide a scientific basis and practical pathway for the sustainable utilization of coal liquefaction residues in asphalt mixtures.

## Figures and Tables

**Table 1:** Technical indicators of ICLR, DCLR and limestone fine aggregate.

Item	Test Results			Technology Standard
	Limestone Fine Aggregate	ICLR	DCLR	
Apparent relative density	2.783	2.612	1.208	≥2.60
Solidity/(%)	87	90	76	≥12
Mud content/(%)	0.7	0.1	0.3	≤3
Sand equivalent/(%)	74	93	81	≥60
Angularity/s	41.7	26.2	86.4	≥30
Water absorption rate/%	1.2	1.5	1.9	≤3

**Table 2:** Properties of DCLR.

Technical Indicators	Result	90 Petroleum Asphalt Technical Indicators	Method
Penetration at 25 °C/(0.1 mm)	2~5	80~100	T 0604
Ductility at 10 °C/(cm)	1.9	≥20	T 0605
Softening Point/(°C)	190	≥42	T 0606

**Table 3:** Mechanical properties of ICLR, DCLR and limestone fine aggregate [17].

Item	Result		
	Limestone Fine Aggregate	ICLR	DCLR
Vickers hardness (HV)	246	615	21
Modulus of elasticity (GPa)	81	98	6.83

**Table 4:** Element composition of ICLR, DCLR and limestone fine aggregate [19].

Index	C	H	O	N	S	Si	Ca	Fe	Ti	Al	Mg
ICLR (%)	11.44	1.80	49.43	0.45	7.63	14.84	5.17	1.86	0.71	6.11	0.57
DCLR (%)	75.9	4.31	—	0.35	2.47	—	—	—	—	—	—
limestone Fine aggregate (%)	11.71	—	48.13	—	—	1.12	39.04	—	—	—	—

**Table 5:** Components of ICLR.

Ingredient Category	SiO_2_	Fe_2_O_3_	TiO_2_	Al_2_O_3_	CaO	MgO	Other
ICLR (%)	51.38	3.95	2.03	19.26	10.58	1.63	11.17

Note: The data tested by authors.

**Table 6:** Components of DCLR.

Divided by Four Components of Asphalt	Saturation	Aromatic Components	Colloid	Asphaltene
Test value (%)	0.8	4.4	13.91	66.2
Divided by extracted components	Heavy oil	Asphaltene	Preasphaltene	Tetrahydrofuran insoluble matter
Test value (%)	20~30	20~40	15~30	45

**Table 7:** Components of limestone fine aggregate.

Ingredient Category	CaCO_3_	SiO_2_
Test value (%)	97.6	2.4

**Table 8:** Four components of DCLR after ignition loss at different temperatures.

Category	Sample Quality (g)	Quality After Combustion (g)	Burning Loss (%)	Saturation (%)	Aromatic Components (%)	Colloid (%)	Asphaltene (%)	I_c_
170 °C	18.0884	18.0422	0.26	0.46	2.01	9.13	88.4	7.98
190 °C	21.1102	20.7832	1.55	0.63	2.47	9.20	87.7	7.57
210 °C	19.2810	18.6274	3.39	1.02	3.09	9.29	86.6	7.08
230 °C	20.5633	19.5709	4.83	1.44	3.54	9.42	85.6	6.71

Note: The detection method refers to NB/SH/T 0509-2010 [29]; colloidal instability coefficient I_c_ = (asphaltene + saturated fraction)/(aromatic fraction + colloid).

**Table 9:** ICLR, DCLR and limestone fine aggregate leaching solution test results.

Items	Result	Items	Result
Copper	Not exceeding the standard	Cadmium	Not exceeding the standard
Lead	Not exceeding the standard	Chromium	Not exceeding the standard
Zinc	Not exceeding the standard	Nickel	Not exceeding the standard
Arsenic	Not exceeding the standard	Mercury	Not exceeding the standard
Naphthalene	Not detected	Benzofluoranthene	Not detected
Dihydrophenanthrene	Not detected	Benzo [a] pyrene	Not detected
Peng	Not detected	Benzoanthracene	Not detected
Fluorene	Not detected	Dibenzoanthracene	Not detected
Phenanthrene	Not detected	Indene pyrene	Not detected
Anthracene	Not detected	Benzo [a] pyrene	Not detected
Fluoranthene	Not detected	Benzofluoranthene	Not detected
Pyrene	Not detected	Benzo [a] pyrene	Not detected
Benzoanthracene	Not detected	Total amount of polycyclic aromatic hydrocarbons	Not detected

**Table 10:** Comparison of physical and chemical properties between ICLR, DCLR and limestone fine aggregate.

Physical and Chemical Properties	Comparison Results	Physical and Chemical Properties	Comparison Results
Basic Performance	Similarly, the particle size distribution is concentrated in the range of 0~4.75 mm	Chemical composition	ICLR is similar, DCLR has certain differences
Mechanical Properties	ICLR is similar, with higher hardness and modulus; DCLR has certain differences, with lower hardness and modulus	Crystal Phase	ICLR is similar and contains a significant amount of SiO_2_ crystal structure and amorphous material; DCLR has certain differences, mainly containing aromatic carbon microcrystals and fatty carbon microcrystals
Microscopic Morphology	Similar, with uniformly distributed scale-like structures on the surface	Functional Groups	ICLR is similar to limestone fine aggregate, with high concentration of DCLR functional groups and rich aromatic and cyclic aromatic structures that are easily bound to asphalt
Pore Size Distribution	ICLR and DCLR are similar and have little difference from limestone fine aggregates	Thermal Stability	ICLR is similar to limestone fine aggregate, with little difference in DCLR
Element composition	ICLR is similar, DCLR has certain differences	Environmental characteristics	Similar, both do not pose a risk of leaching toxicity

## Data Availability

The original contributions presented in this study are included in the article. Further inquiries can be directed to the corresponding authors.
